# Multicomponent reactions

**DOI:** 10.3762/bjoc.7.107

**Published:** 2011-07-13

**Authors:** Thomas J J Müller

**Affiliations:** 1 Heinrich-Heine-Universität Düsseldorf, Institut für Makromolekulare Chemie und Organische Chemie, Lehrstuhl für Organische Chemie, Universitätsstr. 1, 40225 Düsseldorf, Germany

Chemistry as a central science is facing a steadily increasing demand for new chemical entities (NCE). Innovative solutions, in all kinds of disciplines that depend on chemistry, require new molecules with specific properties, and their societal consequences are fundamental and pioneering. However, NCE not only demand a realistic structural space but also their feasibility poses challenges to synthetic chemists. Nowadays the question of how to perform a synthesis has become most crucial.

What is the ideal synthesis [1–2]? Certainly it should be simultaneously simple, safe, short, selective, high yielding, environmentally benign, based on readily available starting materials, and highly diverse. Additionally, the criterion of selectivity has to be matched with increasing significance economical and ecological aspects. In particular multicomponent reactions (MCR) [3] are masterpieces of synthetic efficiency and reaction design. These one-pot processes consist of concatenations of elementary organic reactions under similar conditions. Most interestingly, multicomponent reactions have accompanied the field of organic chemistry since the early days, particularly in heterocyclic chemistry, but have not been recognized as a fundamental principle until Ugi's groundbreaking extension of the Passerini reaction and the conclusions he drew from this.

Now the major conceptual challenge comprises the engineering of novel types of MCR. Most advantageously and practically, MCR can often be extended into combinatorial, solid phase or flow syntheses promising manifold opportunities for developing novel lead structures of active agents, catalysts and even novel molecule-based materials.

This Thematic Series on multicomponent reactions represents a snapshot of a highly dynamic field and spans a broad range, from recent advances in isonitrile-based MCR to transition metal catalysis in MCR; from peptidic and depsi-peptidic to heterocyclic structures; from reactivity-based to property-based concepts. The sympathetic reader, expert or newcomer, will find a tremendous degree of dynamic and exciting new results in this compilation of multicomponent reaction chemistry.

As the guest editor of this Thematic Series I am very grateful to all authors for their excellent contributions and, in particular, to the staff of the Beilstein-Institut for their support and professional realization.

Thomas J. J. Müller

Düsseldorf, July 2011
